# The Peptidylarginine Deiminase Inhibitor Cl-Amidine Suppresses Inducible Nitric Oxide Synthase Expression in Dendritic Cells

**DOI:** 10.3390/ijms18112258

**Published:** 2017-10-27

**Authors:** Byungki Jang, Akihito Ishigami, Yong-Sun Kim, Eun-Kyoung Choi

**Affiliations:** 1 Ilsong Institute of Life Science, Hallym University, Anyang, Gyeonggi-do 14066, Korea; jang@hallym.ac.kr (B.J.); yskim@hallym.ac.kr (Y.-S.K.); 2 Molecular Regulation of Aging, Tokyo Metropolitan Institute of Gerontology, Tokyo 173-0015, Japan; ishigami@tmig.or.jp; 3 Department of Microbiology, College of Medicine, Hallym University, Chuncheon, Gangwon-do 24252, Korea; 4 Department of Biomedical Gerontology, Graduate School of Hallym University, Chuncheon, Gangwon-do 24252, Korea

**Keywords:** Cl-amidine, peptidylarginine deiminase, inducible nitric oxide synthase, dendritic cells, inflammatory diseases

## Abstract

The conversion of peptidylarginine into peptidylcitrulline by calcium-dependent peptidylarginine deiminases (PADs) has been implicated in the pathogenesis of a number of diseases, identifying PADs as therapeutic targets for various diseases. The PAD inhibitor Cl-amidine ameliorates the disease course, severity, and clinical manifestation in multiple disease models, and it also modulates dendritic cell (DC) functions such as cytokine production, antigen presentation, and T cell proliferation. The beneficial effects of Cl-amidine make it an attractive compound for PAD-targeting therapeutic strategies in inflammatory diseases. Here, we found that Cl-amidine inhibited nitric oxide (NO) generation in a time- and dose-dependent manner in maturing DCs activated by lipopolysaccharide (LPS). This suppression of NO generation was independent of changes in NO synthase (NOS) enzyme activity levels but was instead dependent on changes in inducible NO synthase (iNOS) transcription and expression levels. Several upstream signaling pathways for iNOS expression, including the mitogen-activated protein kinase, nuclear factor-κB p65 (NF-κB p65), and hypoxia-inducible factor 1 pathways, were not affected by Cl-amidine. By contrast, the LPS-induced signal transducer and the activator of transcription (STAT) phosphorylation and activator protein-1 (AP-1) transcriptional activities (c-Fos, JunD, and phosphorylated c-Jun) were decreased in Cl-amidine-treated DCs. Inhibition of Janus kinase/STAT signaling dramatically suppressed iNOS expression and NO production, whereas AP-1 inhibition had no effect. These results indicate that Cl-amidine-inhibited STAT activation may suppress iNOS expression. Additionally, we found mildly reduced cyclooxygenase-2 expression and prostaglandin E2 production in Cl-amidine-treated DCs. Our findings indicate that Cl-amidine acts as a novel suppressor of iNOS expression, suggesting that Cl-amidine has the potential to ameliorate the effects of excessive iNOS/NO-linked immune responses.

## 1. Introduction

Peptidylarginine deiminase (PAD) enzymes mediate the citrullination process—which involves the conversion of arginine to citrulline on a protein or peptide but not to free arginine—through the generation of an active site cleft via conformational changes in the presence of high calcium concentrations both in vivo and in vitro [1]. Five PAD (1–4 and 6) enzymes have been identified in humans and rodents and share 70–95% amino acid identity; the calcium binding sites show very high levels of conservation, with the exception of those of PAD6 [1,2].

Citrullination, a class of post-translational modifications, plays key roles in chromatin remodeling, gene transcription, conformational stability, the generation of autoantibodies to self-proteins, and neutrophil extracellular trap (NET) formation. Consequently, citrullination has been linked to autoimmune diseases, inflammatory diseases, cancer, and neurodegeneration [2,3,4,5]. For example, citrullination of myelin basic protein (MBP), a major protein in the myelin sheath, results in open confirmation, rapid degradation by proteases, and less compact protein-lipid interactions, which are associated with a disorganized myelin structure. Notably, citrullinated MBP accounts for 45% of the total MBP in multiple sclerosis (MS) patients and for more than 80% in fulminant MS patients, whereas 20% of MBP is citrullinated in healthy brain and other neurological disorders [2]. Therefore, citrullinated MBP has been suggested as a hallmark of central nervous system demyelination in MS. Rheumatoid arthritis (RA) is characterized by a chronic inflammation of synovial joints with high amounts of citrullinated proteins and infiltration of inflammatory cells, including PAD2/PAD4-expressing macrophages, neutrophils, and dendritic cells [2,6]. Citrullinated proteins are a source of autoantigens that are responsible for the production of anti-citrullinated protein antibodies (ACPAs). ACPAs are detected in 60–80% of RA patients with a specificity of 85–99%; therefore, they are a useful diagnostic marker for RA. Moreover, immune complexes containing ACPA and citrullinated proteins stimulate a cytokine release, and glycosylation of ACPA leads to the differentiation of osteoclasts and bone loss [6,7].

A variety of PAD inhibitors have been shown to directly bind the enzyme active site. Currently, PAD inhibitors are widely studied as potential anti-cancer and anti-inflammatory drugs, and they can dramatically attenuate the disease course, severity, and clinical manifestations in many disease models, including collagen-induced arthritis [8], MS [9], colitis [10,11], atherosclerosis [12], lupus [13], kidney injury [14], hypoxic ischemia [15], and cancer [11,16]. The pan-PAD inhibitor Cl-amidine was designed to irreversibly inhibit PADs through covalent modification at the enzyme active site [17], and its therapeutic potential has been demonstrated in the aforementioned disease models. Recently, Cl-amidine was also shown to modulate dendritic cell (DC) functions, such as cytokine production, antigen presentation, and T-cell proliferation capacity, in vitro and in vivo [18].

In the present study, we found that Cl-amidine acts as a suppressor of inducible nitric oxide synthase (iNOS), leading to decreased NO (represented without a dot for the unpaired electron) production in DCs. NO is a gaseous free radical and is derived from free l-arginine catalyzed by the NOS family, which is divided into three types: calcium (Ca^2+^)-dependent constitutive neuronal NOS (nNOS), endothelial NOS (eNOS), and Ca^2+^-insensitive iNOS. Constitutive NOS induces low levels of NO, whereas iNOS produces high levels of NO for prolonged periods of time. The expression of iNOS primarily depends on pro-inflammatory molecules (signals) and pathogens, and this enzyme is therefore generally associated with the immune system [19,20]. NO modulates a number of physiological and pathophysiological processes, including inflammation, neurotransmission, platelet aggregation, vascular relaxation, and defense against pathogens [19,21]. Therefore, iNOS and NO have been linked to many inflammatory diseases, including cardiovascular diseases, septic shock, asthma, RA, and chronic obstructive pulmonary disease [21,22,23]. For these reasons, selective inhibition of iNOS is a promising approach for the treatment of various inflammatory diseases.

Our findings suggest that the suppression of NO generation underlies the therapeutic effects of Cl-amidine in a number of diseases, and that Cl-amidine may have therapeutic benefits for the treatment of iNOS/NO-associated disorders.

## 2. Results

### 2.1. Cl-Amidine Suppresses NO Generation in Lipopolysaccharide-Stimulated Dendritic Cells

**Figure 1:**
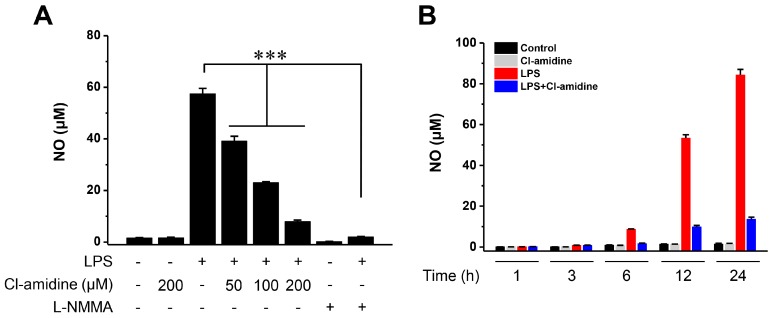
Cl-amidine suppresses nitric oxide (NO) generation in lipopolysaccharide (LPS)-treated dendritic cells (DCs). (**A**) DCs (5 × 10^5^ cells) were stimulated with LPS (0.1 μg/mL), with or without Cl-amidine (50, 100, or 200 μM), for 24 h. l-NMMA (500 μM) was used as a positive control for nitric oxide synthase (NOS) inhibition. (**B**) DCs (1 × 10^6^ cells) were stimulated with LPS (0.1 μg/mL), with or without Cl-amidine (200 μM), for the indicated lengths of time. Quantitative NO levels are represented by bars (mean ± SD; *n* = 3). *** *p* < 0.001.

During LPS-induced DC maturation, we determined NO levels in the absence or presence of Cl-amidine. In DCs treated with Cl-amidine (50, 100, or 200 μM), LPS-induced NO production was significantly reduced by up to 30%, 60%, and 85%, respectively (Figure 1A). The pan-NOS inhibitor N^G^-methyl-l-arginine (l-NMMA) was used as a positive control and completely inhibited NO production. We also found that the decreased NO production induced by Cl-amidine occurs at an early time point (6 h) and in a time-dependent manner (Figure 1B). These results suggest that Cl-amidine acts as a suppressor of NO production.

### 2.2. NOS Activity Is not Inhibited by Cl-Amidine

**Figure 2:**
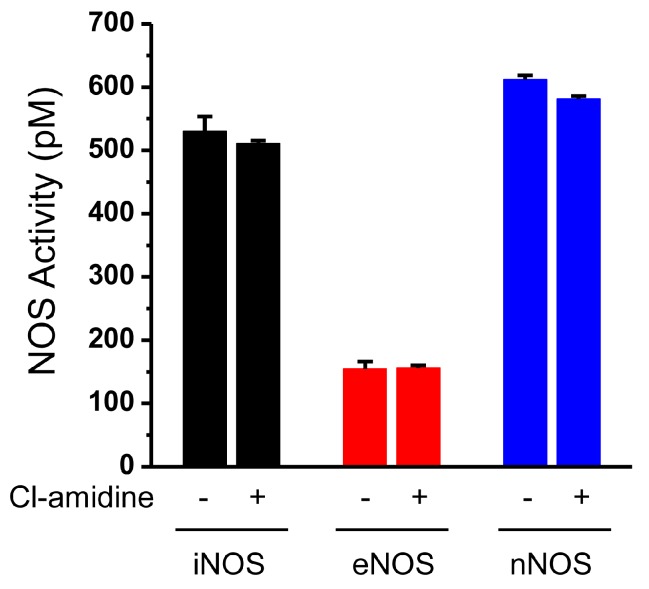
Cl-amidine does not directly inhibit NOS activity. Recombinant NOS (180 ng inducible NO synthase (iNOS); 210 ng endothelial NOS (eNOS); and 420 ng nNOS) was preincubated with or without Cl-amidine (200 μM) for 1 h, and then activity was measured for 1 h. Quantitative levels are represented by bars (mean ± SD; *n* = 3).

Next, we determined whether the decreased NO production we observed was caused by Cl-amidine-induced inhibition of NOS activity. Recombinant NOS enzymes (iNOS, eNOS, and nNOS) were pretreated with Cl-amidine, and we then measured NOS activity. As shown in Figure 2, Cl-amidine had no effect on the enzymatic activity of any NOS enzymes we tested. These results indicate that the inhibition of NO production by Cl-amidine likely occurs through the misregulation of an upstream signaling pathway for NO production.

### 2.3. Cl-Amidine Suppresses iNOS Expression

**Figure 3:**
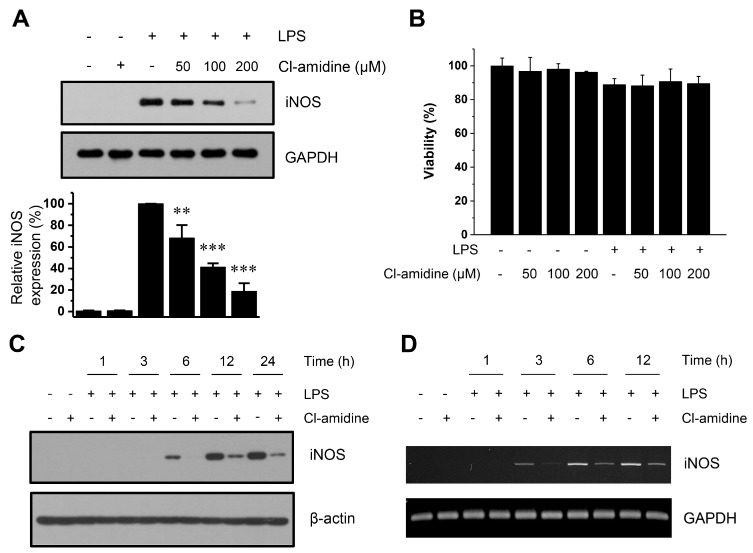
Cl-amidine suppresses iNOS induction in LPS-treated DCs. LPS (0.1 μg/mL)-treated or -untreated DCs (5 × 10^6^ cells) were cultured in the absence or presence of Cl-amidine (50, 100, or 200 μM) for 24 h. (**A**) iNOS expression was detected by Western blotting with an anti-iNOS antibody. Anti-glyceraldehyde 3-phosphate dehydrogenase (GAPDH) was used as the loading control. Bottom panel: relative intensity of iNOS in upper panels with loading controls using three individuals of each group. Error bars represent SD. ** *p* < 0.05, *** *p* < 0.001. (**B**) Cell viability was measured using a CCK-8 staining. (**C**) In the absence or presence of Cl-amidine (200 μM), DCs (4 × 10^6^ cells) were incubated with LPS (0.1 μg/mL) for the indicated lengths of time. iNOS expression was detected by Western blotting with an anti-iNOS antibody. β-actin was used as the loading control. (**D**) In the absence or presence of Cl-amidine (200 μM), DCs (5 × 10^6^ cells) were incubated with LPS (0.1 μg/mL) for the indicated lengths of time. mRNA levels of *iNOS* and *GAPDH* were analyzed by RT-PCR.

In DCs, iNOS is responsible for NO production. Therefore, we measured iNOS expression during DC maturation induced by LPS in the absence or presence of Cl-amidine. In the DCs that were treated with 50 μM, 100 μM, and 200 μM of Cl-amidine, LPS-induced iNOS expression was markedly suppressed by ~30%, 60%, and 80%, respectively (Figure 3A), in a dose-dependent manner. To confirm whether decreased iNOS expression was due to the cell death, we analyzed cell viability using Cell Counting Kit-8 (CCK-8) staining. No significant decrease in viability was observed in our experimental conditions (Figure 3B). Similar to our NO production results, iNOS expression was suppressed at an early time point (6 h) by Cl-amidine (Figure 3C). These results indicate that the down-regulation of NO production by Cl-amidine treatment is caused by suppression of iNOS expression.

Next, we used RT-PCR to investigate whether Cl-amidine affects the production of *iNOS* mRNA transcripts. As shown in Figure 3D, Cl-amidine affected transcription of the *iNOS* gene at an early time point (3 h). Taken together, these findings suggest that the pan-PAD inhibitor Cl-amidine is a candidate for suppression of iNOS.

### 2.4. Cl-Amidine Does not Affect Mitogen-Activated Protein Kinases (MAPKs), Nuclear Factor-κB p65 (NF-κB p65) Signaling, or Hypoxia-Inducible Factor 1α (HIF-1α) Expression but Instead Attenuates STAT Activation

**Figure 4:**
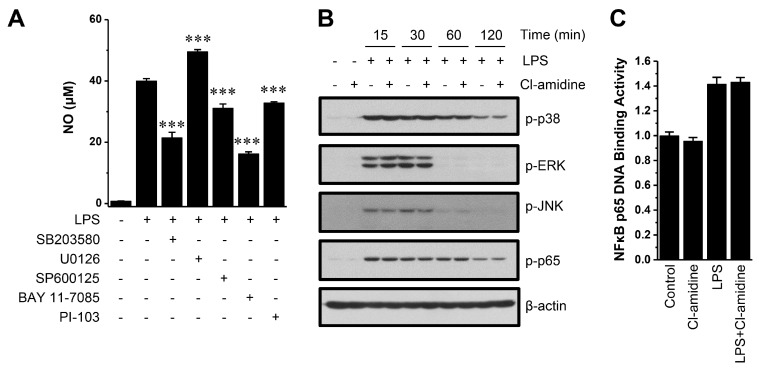

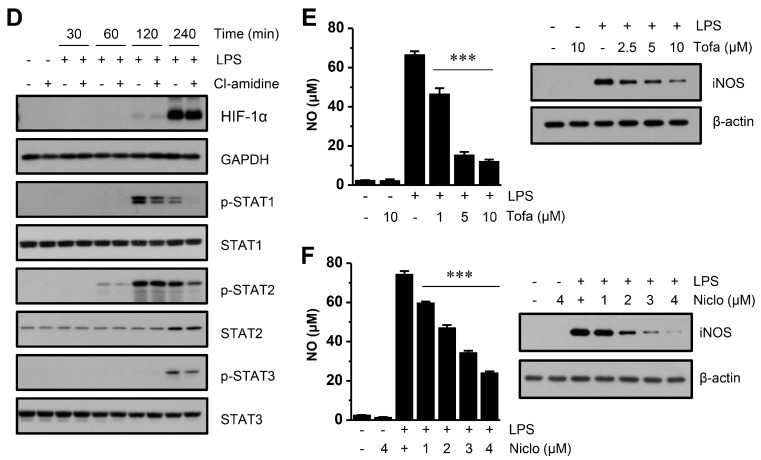
Cl-amidine does not affect MAPK/NF-κB signaling but attenuates STAT activation. (**A**) After pretreatment with SB203580 (20 μM; p38 inhibitor), U0126 (20 μM; ERK inhibitor), SP600125 (20 μM; JNK inhibitor), BAY 11-7085 (20 μM; NF-κB and IκBα inhibitor), or PI-103 (5 μM; PI3K inhibitor) for 2 h, DCs (5 × 10^5^ cells) were incubated with LPS for 24 h. NO was measured by ELISA at 540 nm (mean ± SD; *n* = 3). *** *p* < 0.001. (**B**–**D**) In the absence or presence of Cl-amidine (200 μM), DCs (4 × 10^6^–8 × 10^6^ cells) were incubated with LPS (0.1 μg/mL) for the indicated lengths of time. Expression levels of phosphorylated (p-) p38, ERK, JNK, p65 (**B**), HIF-1α, STAT1, and STAT3 (**D**) were probed by Western blotting with appropriate antibodies. β-actin and GAPDH were used as the loading controls. (**C**) After DCs were activated with LPS for 1 h, DNA binding activity of NF-κB p65 was measured by ELISA as described in the Materials and Methods. (**E**,**F**) After pretreatment with tofacitinib (**E**: Tofa; JAK inhibitor) or niclosamide (**F**: Niclo; STAT inhibitor) for 1.5 h, NO production and iNOS expression were measured by ELISA (**left** panel) and Western blotting (**right** panel). *** *p* < 0.001.

The expression of iNOS is regulated by multiple upstream signaling pathways, including MAPK, NF-κB, phosphatidylinositol 3-kinase (PI3K), STAT-1, and AP-1 [23,24,25]. To determine whether the MAPK, NF-κB, or PI3K pathways are involved in NO generation, we first assessed NO production after exposure to SB203580 (a p38 inhibitor), U0126 (an MAPK/extracellular signal-regulated kinase (ERK)1/2 inhibitor), SP600125 [a C-Jun N-terminal kinase (JNK) inhibitor], BAY 11-7085 (an NF-κB, IκBα inhibitor), and PI-103 (a PI3K inhibitor). NO generation was significantly attenuated by the p38, JNK, NF-κB, and PI3K inhibitors (~47%, ~22%, ~60%, and ~18%, respectively), whereas the inhibitor U0126 enhanced NO generation (Figure 4A). Unchanged MAPK phosphorylation, nuclear translocation of NF-κB p65 and phosphorylation of IκBα by Cl-amidine have been reported previously [18]. Here, we confirmed that Cl-amidine had no effect on the phosphorylation of MAPKs or NF-κB p65 (Figure 4B). Next, we examined whether Cl-amidine affects the DNA binding activity of NF-κB p65. Enhanced DNA binding activity of NF-κB p65 by LPS was not changed by Cl-amidine treatment (Figure 4C). Hypoxia-inducible factor-1α (HIF-1α) can stimulate iNOS expression, but its expression was also unchanged (Figure 4D). Interestingly, LPS-induced STAT1, STAT2, and STAT3 phosphorylation was decreased by Cl-amidine treatment for 2 h and/or 4 h (Figure 4D). Next, we confirmed that the JAK and STAT inhibitors effectively down-regulated iNOS expression. The JAK inhibitor tofacitinib and STAT inhibitor niclosamide dramatically decreased LPS-induced NO production and iNOS expression (Figure 4E,F). These results indicate that decreased STAT phosphorylation by Cl-amidine preferentially suppressed iNOS expression, resulting in reduced production of NO in DCs.

### 2.5. Cl-Amidine Impairs AP-1 Family Member Activity

**Figure 5:**
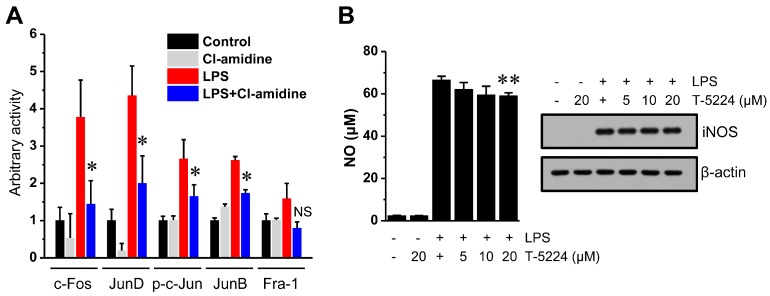
Cl-amidine impairs LPS-induced AP-1 family activity. (**A**) Nuclear proteins (10 μg) isolated from DCs (1 × 10^7^ cells) were stimulated with LPS (0.1 μg/mL) with or without Cl-amidine (200 μM) for 1 h, and then c-Fos, JunD, p-c-Jun, JunB, and Fra-1 activity was measured as described in the Materials and Methods (mean ± SD; *n* = 3). * *p* < 0.05. NS, not significant. (**B**) After DCs (5 × 10^5^ cells or 5 × 10^6^ cells) were incubated with the AP-1 inhibitor T-5224 for 2 h, cells were stimulated with LPS for 24 h, and then NO production (**left** panel) and iNOS expression (**right** panel) were assessed by ELISA and Western blotting. ** *p* < 0.01.

Next, we examined whether Cl-amidine can affect the activity of the AP-1 family, a candidate regulatory factor for iNOS expression. When AP-1 dimers are activated, they bind to a specific double-stranded DNA sequence containing the TPA-responsive element (5′-TGAGTCA-3′), and this AP-1 family activity also regulates iNOS expression [24]. Therefore, we examined whether Cl-amidine regulates LPS-induced activation of AP-1 using nuclear extract. As shown in Figure 5, the expression levels of the LPS-induced AP-1 family members (c-Fos, JunD, p-c-Jun, and JunB) were significantly decreased by Cl-amidine treatment.

In addition, we determined whether the inhibition of AP-1 activity can decrease NO production and iNOS expression. As shown in Figure 5B, treatment with the AP-1 inhibitor T-5224 (20 μM) slightly inhibited NO production (~11%) but not iNOS expression. Moreover, the other AP-1 inhibitor, SR113201, had no effect on NO production. These results indicate that AP-1 activity may not be involved in the regulation of iNOS expression in LPS-activated DCs.

### 2.6. Ineffective Regulation of Cyclooxygenase 2 (COX-2) Expression and Prostaglandin E2 (PGE2) Production due to Cl-Amidine Treatment

**Figure 6:**
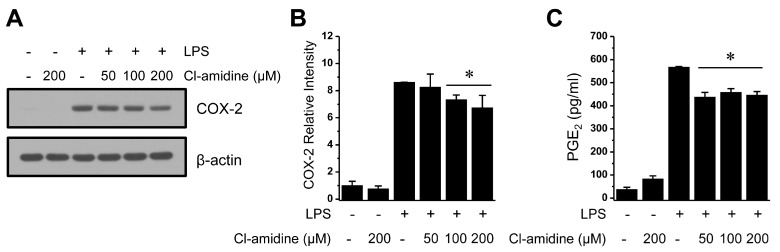
Mild suppression of COX-2 expression and PGE_2_ production by Cl-amidine treatment. (**A**) LPS (0.1 μg/mL)-treated or -untreated DCs (1 × 10^7^ cells) were cultured in the absence or presence of Cl-amidine (50, 100, and 200 μM) for 24 h. COX-2 expression was measured by Western blotting with an anti-COX-2 antibody. β-actin was used as the loading control. (**B**) Columns represent the relative intensity of COX-2 from three independent experiments. (**C**) DCs (1 × 10^5^ cells) were stimulated with LPS (0.1 μg/mL), with or without Cl-amidine (50, 100, or 200 μM), for 24 h. PGE_2_ production was measured as described in the Materials and Methods (mean ± SD; *n* = 3). * *p* < 0.05.

PGE_2_ is a major inflammatory mediator along with NO and is derived from cyclooxygenase 2 (COX-2). Inflammatory stimuli elicit the synthesis of iNOS and COX-2 proteins over similar time courses, and these two proteins also interact [26,27,28]. To investigate whether Cl-amidine affects COX-2 expression and PGE_2_ production, we measured COX-2 expression by Western blotting with an anti-COX-2 antibody and PGE_2_ levels using ELISA in LPS-stimulated DCs in the absence or presence of Cl-amidine. Expression of the COX-2 protein was down-regulated by ~20% in the presence of Cl-amidine treatment compared with the LPS alone treatment (Figure 6A,B), concomitant with a similar reduction in PGE_2_ formation (Figure 6C). This result indicates that Cl-amidine also exerts a mild effect on COX-2 induction and PGE_2_ production.

## 3. Discussion

Recently, PAD enzymes and protein citrullination have been reported to possess a variety of physiological activities, such as tumorigenesis, bactericidal effects, and immunomodulatory effects [5,18,29], suggesting that the inhibition of PAD is a potential therapeutic strategy for the treatment of various disease models. Indeed, the PAD inhibitor Cl-amidine abrogates citrullinated, histone-linked NET formation in vitro and in vivo [3,13], enhances p53-targeted gene expression [5], and regulates the function of immune cells such as T cells and DCs [18]. In the present study, we found that Cl-amidine acts as a potential inhibitor for NO production and iNOS expression (Figure 1 and Figure 3). NO is involved in NET activity, autoimmune diseases, and inflammatory diseases [23,30,31,32,33]. Therefore, it is also possible that the suppression of NO production by Cl-amidine, as described here, might attenuate NO/iNOS-involved disease phenotypes.

NETs have roles in many infectious and noninfectious diseases, including lupus, RA, and cancer [34,35]. NETs are highly decondensed chromatin structures containing histones, protease, and extracellular DNA, which can result in suicidal NETosis or vital NETosis [29,34,35]. The generation of NETs primarily protects the host from infection, but reducing NETs protects against tissue injury [34,36]. PAD4 deficiency and Cl-amidine treatment block NET formation and its release [29,37]. Moreover, exogenous NO promotes NET release, which is blocked by the reactive oxygen species scavenger N-acetyl cysteine [38]. In addition, NO is produced during NET release, and the iNOS inhibitor l-N^G^-nitroarginine methyl ester (l-NAME) reduces NET formation [39,40]. Therefore, we hypothesized that reduced NET formation by Cl-amidine treatment not only blocked histone citrullination but also inhibited NO generation by suppressing iNOS expression. In other words, reduced disease activity by Cl-amidine treatment may be attributed to PAD inactivation with reduced NO generation in a number of diseases, such as lupus, MS, and RA [41].

The expression of iNOS is regulated by multiple upstream signaling pathways in mouse macrophages and DCs, including MAPK, NF-κB, STAT, PI3K, HIF-1α, and the AP1 family [23,24,25]. The inhibition of the MAPK, NF-κB p65, and PI3K signaling pathways inhibited NO production (Figure 4A). The p38 (SB203580) and NF-κB p65 (BAY 11-7085) inhibitors suppressed ~50% of NO production compared with controls. The JNK and PI3K inhibitors were less effective, reducing NO production by ~22% and ~18%, respectively. Both a PI3K deficiency and a PI3K inhibitor impaired NO generation in peritoneal macrophages [42]. By contrast, it was found that two PI3K inhibitors, wortmannin and LY294002, actually increased iNOS and NO production in RAW264.7 macrophages [43]. This discrepancy may have been due to the different types of cells used in each set of experiments. Altogether, our observations indicate that multiple signaling pathways are involved in NO generation in DCs. Interestingly, the ERK inhibitor U0126 enhanced NO production (Figure 4A). U126 was previously shown to enhance NO production in RAW264.7 macrophages [44]. Therefore, ERK may negatively regulate NO production in LPS-stimulated DCs. Because Cl-amidine had no effect on MAPK and NF-κB p65 signaling pathways (Figure 4B) and the DNA-binding activity of NF-κB p65 (Figure 4C), these signaling pathways are not likely associated with Cl-amidine-mediated iNOS suppression, although the MAPK and NF-κB p65 signaling pathways are involved in the regulation of iNOS expression.

The PAD inhibitor BB-Cl-amidine {N-[(1S)-1-(1H-benzimidazol-2-yl)-4-[(2-chloro-1-iminoethyl)amino]butyl]-[1,1′-biphenyl]-4-carboxamide} inhibited LPS-induced expression of interleukin (IL)-1β and tumor necrosis factor α (TNFα) by preventing nuclear translocation of NF-κB p65 in neutrophils [45]. Interestingly, citrullination of NF-κB p65 by LPS-mediated PAD4 enzyme activity enhanced its interaction with importin α3 and nuclear translocation. Similarly, PAD4 promoted nuclear translocation of NF-κB p65 in renal proximal tubules [46]. By contrast, Cl-amidine had no impact on LPS-induced phosphorylation and DNA binding activity of NF-κB p65 (Figure 4B,C) and degradation of IκBα in DCs, although Cl-amidine inhibits LPS-induced cytokine production of IL-1β, TNFα, and IL-12p70 [18]. Because PAD activity is effectively inhibited by both BB-Cl-amidine and Cl-amidine, we hypothesized that PAD-targeted proteins and their function are different in each cell type, i.e., histone H3 citrullination is involved in NET formation in neutrophils and regulates gene expression in cancer cells [3,5,13]. Moreover, LPS promotes citrullination [18], and histone H3 is predominantly citrullinated in DCs. It is still unclear whether histone H3 citrullination is important during DC maturation, including gene expression, which should be further investigated in detail.

JAK/STAT were implicated in NO generation in a variety of cells [47,48,49]. As JAK undergoes transphosphorylation and, subsequently, phosphorylate STATs, activated STAT proteins dimerize and translocate into the nucleus, where they activate or repress target gene promoters [50]. In this study, Cl-amidine down-regulated STAT phosphorylation (Figure 4D). Involvement of STAT signaling in iNOS expression was also confirmed by treatment with the JAK inhibitor tofacitinib and the STAT inhibitor niclosamide (Figure 4E,F). Although AP-1 activity, a potential regulator for iNOS expression, was significantly decreased by Cl-amidine, both iNOS expression and NO production in DCs were not affected by AP-1 inhibition (T-5224) (Figure 5). Therefore, Cl-amidine-mediated suppression of iNOS expression may be due to the suppression of STAT phosphorylation but not the MAPK, NF-κB, HIF-1α, and AP-1 signaling pathways.

Inflammatory stimuli elicit both NO and PGE_2_ production through the induction of iNOS and COX-2 over similar time courses. More specifically, NO enhances PGE_2_ production through S-nitrosylation of COX-2 [28]. Our data showed that LPS-induced COX-2 expression and PGE_2_ production still maintained ~80% of their normal expression levels by Cl-amidine (Figure 6). Therefore, our results indicate that Cl-amidine selectively inhibits iNOS induction rather than COX-2 induction signaling. It is possible that a combination of Cl-amidine with a COX-2 inhibitor may have synergistic therapeutic benefits in inflammatory diseases.

Physiologically, NO attacks the thiol groups of cysteines on proteins, forms S-nitrosothiols, and reacts with superoxide anions radical (O_2_^−^) to generate peroxynitrite (ONOO^−^), which can modify proteins (e.g., tyrosine nitration) [23]. These modifications are involved in a number of disease models and cellular processes, such as signal transduction and apoptosis [51,52]. Furthermore, ONOO^−^ secreted from DCs can kill tumor cells [53]. Therefore, further studies are needed to determine whether Cl-amidine can reduce S-nitrosothiol levels and nitration formation and modulate associated signaling pathways.

In this study, we determined that Cl-amidine inhibits NO generation by suppressing iNOS transcription in DCs. It is also possible that Cl-amidine may have effects on iNOS expression in other cell types, including macrophages. Indeed, Witalison et al. showed that Cl-amidine suppressed iNOS induction in mouse macrophages [54]. NO also plays an important role in epithelial cells and endothelial cells, and further studies will be needed to determine whether Cl-amidine has an effect on these cell types.

In summary, iNOS expression and NO production are reduced in DCs by the PAD inhibitor Cl-amidine through the repression of STAT activities. We hypothesized that Cl-amidine may downregulate NO production via modulation of iNOS expression in the other cell types, including macrophages. Taken together, these findings indicate that Cl-amidine could improve excessive NO-mediated inflammatory responses and damage in certain disease states. Therefore, PAD inhibitors should be further examined to determine whether they have potential therapeutic effect on the disease(s) associated with iNOS activity and/or NO production.

## 4. Materials and Methods

### 4.1. Chemicals and Reagents

Cl-amidine (Calbiochem, Darmstadt, Germany), the TLR4 agonist lipopolysaccharide (ultrapure LPS; Invivogen, San Diego, CA, USA), SB203580, U0126, SP600125, BAY 11-7085, PI-103 (Adooq, Irvine, CA, USA), Tofacitinib, niclosamide (Tocris, Bristol, UK), and T-5224 (APExBIO, Houston, TX, USA) were purchased commercially. Unless otherwise stated, all chemicals and reagents were purchased from Sigma-Aldrich (St. Louis, MO, USA). Ten or twenty millimoles stock solution of Cl-amidine was dissolved in phosphate buffer solution (PBS), and other inhibitors were dissolved in DMSO. PBS or DMSO were used as vehicle controls in each experiment.

### 4.2. Cell Culture

C57BL/6 female mice (6–8 weeks of age) were purchased commercially (Orient bio, Seongnam, Korea). Animal experiments and research protocols were approved by the Institutional Animal Care and Use Committee of Hallym University (HMC2015-0-1130-16; 23 December 2015). DCs were cultured as described previously [16]. Briefly, bone marrow cells from the femurs and tibiae of C57BL/6 female mice (6–8 weeks) were cultured in RPMI-1640 (Thermo Fisher Scientific, Waltham, MA, USA) containing 10% fetal bovine serum (Biowest, Riverside, MO, USA), 10 mM HEPES buffer (pH 7.4, Sigma-Aldrich), 2 mM l-alanyl-l-glutamine dipeptide (Sigma-Aldrich), 50 μM 2-mercaptoethanol, MEM non-essential complements (Sigma-Aldrich), 20 ng/mL recombinant mouse granulocyte-macrophage colony stimulating factor (JW CreaGene, Seongnam, Korea), 0.5 ng/mL interleukin (IL)-4 (JW CreaGene), 100 units/mL penicillin, and 100 μg/mL streptomycin at 37 °C in a 5% CO_2_ incubator. Fresh medium was added on days 4 and 7, and all experiments were performed after 8 days of culture. The samples had more than 80% CD11c^+^ cells, as determined by flow cytometry.

### 4.3. NO Detection and NOS Activity

The culture medium was obtained from LPS (0.1 μg/mL)- or vehicle-stimulated DCs (5 × 10^5^ or 1 × 10^6^ cells) in the absence or presence of Cl-amidine (50–200 μM). The levels of NO in the culture medium were measured by ELISA according to the manufacturer’s instructions (Intron Biotechnology, Seongnam, Korea). To assay NOS activity, recombinant NOS (180 ng iNOS; 210 ng eNOS; 420 ng nNOS; Enzo Life Sciences, New York, NY, USA) was incubated with or without Cl-amidine (200 μM) for 1 h, and then activity was measured for 1 h by ELISA, according to the manufacturer’s instructions (Biovision, Milpitas, CA, USA).

### 4.4. Western Blotting Analysis

C57BL/6 DCs were lysed in PRO-PREP lysis buffer (Intron Biotechnology), and protein concentrations were measured using the Bicinchoninic Acid Kit (Thermo Fisher Scientific). Samples (40–50 μg) were loaded into sodium dodecyl sulfate polyacrylamide electrophoresis gels and transferred to polyvinylidene difluoride membranes (Merck-Millipore, Billerica, MA, USA). Rabbit monoclonal anti-phospho-p38 (1:1000), rabbit monoclonal anti-phospho-ERK (1:1000), rabbit monoclonal anti-phospho-JNK (1:1000), rabbit monoclonal anti-phospho-NF-κB p65 (1:1000), rabbit monoclonal anti-phospho-STAT1 (1:1000), rabbit monoclonal anti-phospho-STAT3 (1:1000), rabbit monoclonal anti-STAT1 (1:2000), rabbit monoclonal anti-STAT2 (1:2000), rabbit monoclonal anti-STAT3 (1:2000) (Cell Signaling, Danvers, MA, USA), and rabbit polyclonal anti-phospho-STAT2 antibodies (Merck-Millipore; 1:1000) were purchased and used in the Western blotting experiments. Mouse monoclonal anti-HIF-1α (1:1000; Abcam, Cambridge, MA, USA), mouse monoclonal anti-β-actin (1:10,000; Sigma-Aldrich), mouse monoclonal anti-GAPDH (1:10,000; BETHYL, Montgomery, TX, USA), rabbit polyclonal anti-iNOS (1:1000; Santa Cruz Biotechnology, Santa Cruz, CA, USA), and goat polyclonal anti-COX-2 (1:1000; Santa Cruz Biotechnology) antibodies were used in the Western blotting experiments. The primary antibodies were detected with appropriate horseradish peroxidase-conjugated goat anti-mouse, rabbit anti-goat, or goat anti-rabbit secondary antibodies (1:5000; Cell Signaling or Merck-Millipore). Chemiluminescent signals were detected on X-ray film (Agfa HealthCare, Mortsel, Belgium) using ECL Western blotting detection reagents (ATTO, Tokyo, Japan).

### 4.5. Semi-Quantitative Reverse Transcriptase Polymerase Chain Reaction (RT-PCR)

DCs were stimulated with LPS (0.1 μg/mL) with or without Cl-amidine (200 μM) for the indicated times, and then total RNA was extracted using the Ribospin RNA extraction kit following the manufacturer’s instructions (GeneAll, Seoul, Korea). The cDNA synthesis was performed with AMV reverse transcriptase (Promega, Madison, WI, USA) according to the instructions of the manufacturer. RT-PCR was performed using primers specific to the iNOS (600 bp; forward, 5′-GGTATGCTGTGTTTGGCCTT-3′ and reverse, 5′-GCAGCCTCTTGTCTTTGACC-3′) and GAPDH (200 bp; forward, 5′-TGGTATCGTGGAAGGACTCATGAC-3′ and reverse, and 5′-ATGCCAGTGAGCTTCCCGTTCAGC-3′) genes with GoTaq DNA polymerase, according to the manufacturer’s instructions (Promega). The resulting RT-PCR products were separated by gel electrophoresis on a 1.0% agarose gel with the SafeView nucleic acid gel stain (ABM, Richmond, BC, Canada) and then visualized using UV light.

### 4.6. NF-κB p65 Transcription Factor Activity Assay

DCs (8 × 10^6^ cells) were stimulated with LPS (0.1 μg/mL), with or without Cl-amidine (200 μM), for 1 h, and then cell lysates (20 μg) were assessed with the NF-κB p65 Transcription Factor Assay Kit, following the manufacturer’s instructions (Abcam).

### 4.7. Activator Protein-1 (AP-1) Transcription Activity Assay

DCs (1 × 10^7^ cells) were stimulated with LPS (0.1 μg/mL), with or without Cl-amidine (200 μM), for 1 h, and then nuclear proteins were isolated using the Nuclear Extraction Kit, following the manufacturer’s instructions (Abcam). To determine AP-1 family member activity, ten micrograms of total nuclear protein used with the AP-1 Transcription Factor Assay Kit, according to the manufacturer’s instructions (Abcam).

### 4.8. Prostaglandin E_2_ (PGE_2_) Assay

LPS (0.1 μg/mL)-treated or -untreated DCs (1 × 10^5^ cells) were cultured in the absence or presence of Cl-amidine (50, 100, or 200 μM) for 24 h. PGE_2_ production was measured by ELISA, according to the manufacturer’s instructions (Enzo Life Sciences, Farmingdale, NY, USA).

### 4.9. Statistical Analysis

All data are presented as mean ± standard deviation (SD) values. The probability of statistically significant differences between experimental groups was assessed by a two-sample *t* test or by one-way analysis of variance.

## Abbreviations

PAD	Pepitylarginine deiminase
iNOS	Inducible nitric oxide synthase
DC	Dendritic cell
LPS	Lipopolysaccharide
AP-1	Activator protein 1
MAPK	Mitogen-activated protein kinases
HIF-1	Hypoxia-inducible factor 1
NF-κB p65	Nuclear factor-kappaB p65
PI3K	Phosphatidylinositol 3-kinase
JNK	C-Jun N-terminal kinase
IL	Interleukin
COX-2	Cyclooxygenase 2
PGE_2_	Prostaglandin E_2_
JAK	Janus kinase
STAT	Signal transducer and activator of transcription
